# Stable Isotope Analysis (δ^13^C, δ^15^N and δ^18^O) of Killer Whale Dentine: Evaluating Treatment Methods and Establishing Best Practices

**DOI:** 10.1002/rcm.70176

**Published:** 2026-09-15

**Authors:** Maeva Terrapon, Philip S. Hammond, Rona A. R. McGill, Jason Newton, Julie Dougans, Cory J. D. Matthews, Sascha K. Hooker

**Affiliations:** ^1^ Sea Mammal Research Unit University of St Andrews St Andrews UK; ^2^ National Environmental Isotope Facility SUERC East Kilbride UK; ^3^ Fisheries and Oceans Canada Winnipeg MB Canada

**Keywords:** acidification, collagen, decalcification, demineralisation, hydroxyapatite, isotopes, treatment

## Abstract

**Rationale:**

Stable isotope measurements in odontocete teeth can be used to reconstruct lifetime ecology, but some pre‐treatments may introduce bias. Isolating dentine collagen with HCl decalcification, removing the organic matrix or drying dentine prior to the analysis of structural carbonates can introduce unintended variability, limiting comparability among studies and populations. This study tested pre‐treatment methods to quantify their effects.

**Methods:**

Killer whale teeth were sectioned, annual growth layers microdrilled, and powder allocated to treatments. For collagen δ^13^C and δ^15^N, 75 samples were analysed via EA‐IRMS as untreated bulk dentine and after 0.25 N HCl decalcification. For structural carbonate δ^13^C and δ^18^O, treatment with 30% H_2_O_2_ was compared with plasma‐ashing and with no treatment (*n* = 16); and we examined the removal of residual water by comparing samples oven‐dried at 80°C or 100°C with untreated samples (*n* = 10). All samples for carbonate analysis were injected with 104% orthophosphoric acid to generate CO_2_, which was analysed using continuous‐flow IRMS.

**Results:**

HCl decalcification caused mass loss (96.6% ± 2.0%) but yielded collagen of an acceptable quality (C:*N* = 3.3 ± 0.2). Compared with untreated dentine, collagen δ^13^C decreased (Δmedian = −0.34‰) while δ^15^N did not differ significantly. Organic‐matrix removal treatments altered both isotopes: plasma‐ashed samples showed large negative shifts compared with untreated samples in δ^13^C (Δmedian = −9.04‰) and δ^18^O (Δmean = −13.48‰) and reduced measurable carbon. H_2_O_2_ treatments lowered δ^13^C relative to untreated samples (Δmean = −0.93‰) but showed no consistent δ^18^O effect. Drying at 80°C minimised δ^13^C and δ^18^O variability.

**Conclusions:**

HCl decalcification is recommended before δ^13^C and δ^15^N collagen analysis of killer whale dentine, or a δ^13^C correction factor (here −0.34‰) should be applied. Treatments were unsuitable when undertaking structural carbonate analyses of hydroxyapatite: we suggest that untreated powder oven‐dried overnight at 80°C should be used, and we emphasise the importance of pre‐treatment tests prior to any new study.

## 1 Introduction

Carbon (δ^13^C), nitrogen (δ^15^N) and oxygen (δ^18^O) isotopes are commonly used as markers to study the diet, movements and habitat use of terrestrial and marine animals. These chemical elements are integrated into the animal's body tissues either from ingestion or from the surrounding environment and can address ecological questions on either short (skin, liver, blubber) or long (bone, teeth, hair) timescales depending on the turnover of the tissue analysed [1]. Stable isotopes in teeth are especially useful to infer lifelong ecology of species which are challenging to observe and sample in the wild, such as large odontocetes that spend most of their time offshore. In comparison with bone, which generally has a homogeneous stable isotope composition, teeth are composed of dentine that builds up continuously through life as well‐defined growth layers. Referred to as growth layer groups (GLGs), these typically accumulate annually from the first year of life and are not altered with time [2].

Dentine is composed of a mineral component (hydroxyapatite, 70%), an organic matrix (mostly collagen, 20%) and water (10%) [3]. Both mineral and organic components can be used for stable isotope analysis. Collagen is produced from dietary proteins, and δ^13^C and δ^15^N from this therefore reflect the ecological niche of the animal [4].

The hydroxyapatite (Ca_10_[PO_4_,CO_3_]_6_[OH,CO_3_]_2_) component, composed of phosphates and structural carbonates, can be analysed for oxygen isotope composition to infer odontocete movements and distribution [5, 6, 7]. δ^18^O in hydroxyapatite is integrated in dentine over time from body water (a combination of metabolic water, ingested water, respired vapour and skin‐absorbed water). It precipitates with constant fractionation in homeothermic animals (between body water and mineral) and is generally influenced by the δ^18^O of marine water, reflecting environmental parameters such as temperature and salinity [5, 8, 9]. δ^18^O in both phosphates and carbonates can be analysed [5], but one advantage of working with carbonates is the additional possibility to obtain carbon isotope values, which can be informative for movement studies [10]. The hydroxyapatite carbon is of dietary origin, but integration pathways differ from collagen carbon. Indeed, whereas carbon in collagen exclusively originates from dietary proteins, carbon in structural carbonates originates from dietary lipids, carbohydrates and proteins [4, 11]. These different pathways result in an isotopic difference between δ^13^C_collagen_ and δ^13^C_structural carbonate (SC)_, which depends on the type of prey consumed (if mostly proteins, then a positive relationship would be expected) [3, 12, 13, 14].

Given the differences in information that can be inferred from stable isotope analysis of either collagen or structural carbonates of hydroxyapatite, it is prudent to isolate each component prior to conducting analyses, or at least to ensure the presence of one component will not have a confounding impact on the isotopic composition measured. To remove the potential contribution of inorganic carbon for δ^13^C and δ^15^N analysis of collagen, acidification with hydrochloric acid (HCl) is recommended for bulk dentine [1, 4]. However, this treatment could unnecessarily impact δ^15^N values [15, 16]. Whether this method is necessary for modern marine mammal dentine is currently unclear. Most published work specifically comparing isotope compositions of untreated and decalcified dentine samples from modern samples found that acidification treatments did not change δ^13^C and δ^15^N isotope values [6, 17, 18], leading to the recommendation that acidification of dentine powder is unnecessary prior to analysis. However, this approach is still widely used in marine mammal isotope work [19, 20, 21, 22, 23, 24, 25, 26]. Variability across published results, as well as the species‐specificity and small sample size of some of these studies, suggests the utility of testing the effect of this treatment prior to δ^13^C and δ^15^N stable isotope analysis.

Treatments to remove the collagenous organic component from teeth have typically used hydrogen peroxide (H_2_O_2_) or sodium hypochlorite (NaOCl). While some studies strongly recommend using H_2_O_2_ instead of NaOCl prior to carbon and oxygen isotope analyses [27, 28], others have found no difference between them [14, 29] and NaOCl is still used [3, 13, 30]. Others have warned that both treatments can induce serious bias, resulting in an isotopic shift due to alteration of biogenic carbonates, addition of exogenous carbonates or inefficient organic removal [31, 32]. Low temperature oxygen plasma‐ashing is another method used in geoscience research to remove organic material from sediments or other mineral structures prior to δ^13^C and δ^18^O carbonate analysis [33, 34]. Plasma‐ashing oxidises organic components under vacuum, using a lit plasma to create CO_2_ with free oxygen radicals. This method efficiently removes organic material while preserving the mineral matrix without leading to an isotopic shift [35, 36], but no published study to date has used this treatment method for isotope analysis of carbonates in dentine hydroxyapatite. Most of the few studies that have measured δ^18^O isotopes in marine mammal hydroxyapatite (dentine or bone) have applied a hydrogen peroxide treatment prior to analysis, with no published data on the impact of this treatment on their samples and resulting isotope values [6, 37, 38]. One study attempted to extract the organic component from killer whale teeth with NaOCl but did not obtain satisfactory results, and hence analysed untreated dentine samples instead [5].

Dentine powder δ^18^O isotope values could also be affected by residual water in the tooth structure or by exchange with atmospheric water during the drilling process, and drying of the powdered samples prior to analysis is therefore recommended [5]. The impact of the drying process on marine mammal dentine has not been widely explored. Lower δ^18^O values than expected were recorded for untreated killer whale dentine powder oven‐dried at 80°C prior to analysis, potentially because the process might not have removed all water [5].

The aim of this study was to compare treatment methods targeting the mineral and organic components of killer whale dentine to inform best practices for future studies. We present an investigation of the isotopic differences between untreated dentine and (i) dentine acidified using HCl to dissolve the structural carbonate, (ii) dentine treated using plasma‐ashing and H_2_O_2_ to remove the collagenous organic matrix and (iii) dentine oven‐dried at 80°C and 100°C to remove residual water prior to analysis of structural carbonates (Table 1).

**TABLE 1:** Comparison of treatment aims for killer whale dentine components, analysed from two different halves of the same tooth.

	Half tooth 1	Half tooth 2
**Half tooth pre‐treatment**	Surface etching (formic acid)	No pre‐treatment
**Analysed isotopes**	Carbon (δ^13^C), Nitrogen (δ^15^N)	Carbon (δ^13^C), Oxygen (δ^18^O)

Treatment aim	Remove inorganic carbonates (*n* = 75)		Remove collagenous organic component (*n* = 16)			Remove residual water (*n* = 10)		
**Treatment type**	Hydro‐chloric acid (HCl)	No treatment	Hydrogen peroxide (H_2_O_2_)	Plasma‐ashing	No treatment	Oven‐drying 80°C	Oven‐drying 100°C	No treatment
**Analysed material**	Collagen	Dentine	Carbonates	Carbonates	Dentine	Dentine	Dentine	Dentine

## 2 Methods

### 2.1 Dentine Powder Preparation

Killer whale teeth from different individuals and locations (*n* = 11, Table 2) were sourced from museum collections or private donations. These were longitudinally sectioned in two halves with a 0.4‐mm water‐cooled diamond‐coated blade on a Buehler IsoMet 1000 precision saw. For each specimen, the surface layer of one of the halves was etched with 10% formic acid to help with GLG identification (defined either as the layer between two grooves formed during the etching process or as the space between two white lines under reflected light for unetched halves). Each GLG was then marked with a scalpel across its entire length, and each half tooth was mounted on a plastic rectangular mount with hot glue to ensure the tooth surface remained stable and flat. Finally, GLGs were drilled separately to a 0.3 mm depth using a NewWave micromill (MicroMill software v.1.3.10.0) with a 0.3 mm drill bit, and dentine powder was stored in pre‐weighed 2 mL polypropylene (PP) centrifuge tubes for analysis.

**TABLE 2:** Killer whale tooth specimens used in this study. Not all specimens were used for all method testing analyses; test numbers represent the number of different GLG samples used from each specimen for each analysis.

Tooth ID	Provenance	Collection year	Collagen tests	Carbonate tests	Drying tests	Provider and specimen ID
OOT02	United Kingdom	2016	5	3	2	National Museums Scotland (Z.2016.118)
OOT03	United Kingdom	1994	14	9	0	National Museums Scotland (Z.2001.7)
OOT04	Brazil	1993	7	4	2	Dr Silvina Botta (Ecomega101)
OOT05	Mozambique	2021	9	0	9	MHNMM (MHNMM‐MM‐001‐2022)
OOT06	Australia	2008	3	0	0	Australian Museum (M.40076)
OOT07	Madagascar	1974	10	0	5	Iziko Museum South Africa (ZM36954)
OOT08	Madagascar	1974	4	0	0	Iziko Museum South Africa (ZM36953)
OOT09	Seychelles	1850	2	0	0	National History Museum UK (NHMUK.GERM.1165c)
OOT10	South Africa	1991	6	0	0	Iziko Museum South Africa (ZM41118)
OOT11	Morocco	2010	4	0	0	Dr Hicham Masski (NA)
OOT12	Hawaii, USA	2008	11	0	0	Dr Kristi West (KW2008010)

### 2.2 Removal of Structural Carbonate (for δ^13^C and δ^15^N Analysis)

Powdered dentine GLG samples (*n* = 75) from acid‐etched teeth (*n* = 11) were used for carbon and nitrogen isotope analysis. For each sampled GLG, 2 mg of untreated powder was weighed into a tin capsule for isotope analysis. The remainder of the powder (20–50 mg depending on GLG size) was treated three times with 1 mL of 0.25 N HCl at room temperature (~12 h per treatment). Between each treatment, vials were centrifuged at 5000 rpm for 5 min to concentrate the collagenous residue at the bottom of each vial, and the supernatant was removed with a pipette. After the third HCl treatment, three 1 h milliQ water rinses were carried out following the same procedure. On completion of the third milliQ water rinse, the supernatant was checked for neutrality using standard pH paper and additional rinses were conducted if neutrality was not reached. Once the final supernatant was removed, the remaining sample material was frozen overnight, freeze‐dried for 48 h to ensure removal of all water, and weighed to calculate the collagen extraction yield.

### 2.3 Removal of the Collagenous Organic Matrix (for δ^13^C and δ^18^O Analysis)

Powdered dentine samples (*n* = 16) obtained from the non‐etched teeth of three different individuals were prepared for δ^13^C and δ^18^O analysis of structural carbonates in dentine hydroxyapatite. Each sample was separated into three sub‐samples of equal weight (7–15 mg, depending on powder availability) to allow comparison of the three treatments targeting removal of the organic matrix (no treatment, plasma‐ashing and H_2_O_2_ treatment).

#### 2.3.1 Plasma‐Ashing

Each sub‐sample was loaded into a small glass petri‐dish and placed in a plasma asher (Emitech K1050X) for 15–20 h in cycles of 3–4 h. Dentine was ashed at 80 W under vacuum (8 × 10^−2^ mbar) and a visible plasma was maintained throughout ashing to ensure efficient removal of the organic matrix. Samples were weighed between each cycle to track loss of organic material and mixed with a clean stainless‐steel spatula to ensure all powder was exposed throughout the process. Plasma‐ashing was continued until the weight difference between two cycles was smaller than 1 mg, or if the weight difference curve across cycles started to flatten out [35]. The remaining dentine powder was stored in a capped glass vial until analysis.

#### 2.3.2 Hydrogen Peroxide (H_2_O_2_)

Each powder sub‐sample was treated for 24 h with 1 mL of 30% H_2_O_2_ at room temperature [6, 37]. Vials were kept open to allow the evolved CO_2_ to escape. All vials were then centrifuged for 5 min at 5000 rpm and rinsed three times for 1 h with milliQ water, the final supernatant was removed with a pipette and the treated wet powder was frozen for 24 h and freeze‐dried for 48 h.

To assess whether the organic matrix was successfully removed by these methods, the concentration of nitrogen in a subset of the treated samples (*n* = 5) was analysed [39]. Nitrogen in dentine is mostly present in collagen, and thus its concentration should greatly decrease in samples treated for the removal of organic components [39]. Nitrogen mass (in percent [%] and in milligrams [mg]) was estimated by loading 2 mg of treated powder into tin capsules and following δ^15^N isotope analysis methods (see below).

### 2.4 Drying Untreated Dentine Powder (for δ^13^C and δ^18^O Analysis)

To investigate the effect of the drying process on δ^13^C and δ^18^O isotopic values, 18 samples from non‐etched teeth were each separated into two 10–15 mg sub‐samples and loaded into seal‐capped glass vials. We used dentine powder from different individuals than those used in the previous treatments because sample material was limited and larger sample mass was required for analysis of the untreated powder. Only two sub‐samples could be tested from each GLG for this analysis.

Matthews et al. [5] suggested that oven‐drying at 80°C might not be sufficient to remove all residual water. We therefore compared oven‐drying of powder samples at 80°C or 100°C with powder samples which were not oven‐dried, using 80°C as the baseline for comparison. Ten samples were each split into two sub‐samples; one was dried at 80°C and the other at 100°C, both overnight. Ten different samples were similarly split into two aliquots; one was dried at 80°C and the other was not dried. One GLG sample yielded enough powder to split into three sub‐samples allowing comparison of all three methods in one sample. Drying at 80°C and 100°C followed the same method: sub‐samples were placed in an uncapped, upright glass vial in the oven (Thermo Electron Heraeus Function Line Incubator) overnight (~14 h) and immediately sealed with a sealed cap after drying.

### 2.5 Stable Isotope Analysis

All isotope results are expressed in parts per thousand (permil (‰)) using delta notation [40] relative to international references (atmospheric N_2_ [air] for nitrogen; Vienna‐PeeDee Belemnite [VPDB] for carbon and oxygen, and Vienna Standard Mean Ocean Water [VSMOW] for oxygen values after conversion).

For δ^13^C and δ^15^N analysis, between 0.1 and 1 mg of collagen (depending on GLG sample size) or approximately 2 mg of untreated powder from each sample was weighed into tin capsules. All samples were combusted in an elemental analyser (Elementar vario PYRO‐Cube) and resulting carbon and nitrogen gases were analysed using an Isotope Ratio Mass Spectrometer (IRMS) (Thermo Fisher Scientific Delta Plus XP) (except for OOT12 samples, which were loaded into a FLASH EA Isolink and analysed on a Delta Q Mass Spectrometer (both Thermo Fisher Scientific)). Results were corrected for linearity (to account for sample weight differences) and instrument drift [41] over 22 h for a typical analytical run with laboratory standards (gel [Fluka‐gelatine], alagel [gel and Sigma‐alanine], glygel [gel and Sigma‐glycine]; repeatedly analysed every 10 samples). Instrument accuracy and precision were estimated across all runs using repeated measures (*n* = 32) of the international standard USGS40 [42](δ^13^C accepted = −26.24‰, δ^13^C measured = −26.39‰ ± 0.08‰; δ^15^N accepted = −4.52‰; δ^15^N measured = −4.62‰ ± 0.14‰), treated as an independent sample.

For δ^13^C and δ^18^O analysis, 5–10 mg (treated for organic matrix removal) or 10–15 mg (untreated) of dentine powder were weighed into seal‐capped glass vials for analysis. Measurements were made on an Analytical Precision AP2003 mass spectrometer equipped with a separate carbonate acid‐injector system, after reaction with 104% orthophosphoric acid at 70°C. Values were calibrated relative to VPDB and corrected with an internal calcite standard (ATC, *n* = 24, δ^13^C = −9.18‰ ± 0.13‰, δ^18^O = −15.04‰ ± 0.15‰), analysed several times across a given run. Samples and standards were digested under identical phosphoric acid reaction conditions. Measured CO_2_ isotope ratios were normalised to VPDB using international calcite standards; therefore, no additional correction for carbonate‐to‐CO_2_ acid fractionation was applied. Additional calcite international standards (IAEA 603 [43](*n* = 12, δ^13^C = 2.50‰ ± 0.16‰, δ^18^O = −2.37‰ ± 0.12‰), IAEA CO‐8 (*n* = 2, δ^13^C = −5.58‰ ± 0.07‰, δ^18^O = −22.93‰ ± 0.20‰), IAEA CO‐9 (*n* = 12, δ^13^C = −46.68‰ ± 0.37‰, δ^18^O = −15.05‰ ± 0.16‰), NBS‐18 (*n* = 4, δ^13^C = −5.05‰ ± 0.23‰, δ^18^O = −23.07‰ ± 0.13‰) [44]) were included in analytical runs and treated as unknown to verify instrument precision. Accuracy was verified by comparing the values obtained above with the accepted value of each international standard, and the differences ranged from 0.04‰ to 0.64‰ for δ^13^C, and from 0‰ to 0.55‰ for δ^18^O. δ^18^O_VPDB_ was then converted to δ^18^O_VSMOW_ following the conversion equation [45]:

$$\delta^{18}O_{\text{VSMOW}}=\delta^{18}O_{\text{VPDB}}\times 1.03086+30.86$$

Both VPDB and VSMOW international references are used for oxygen isotope reporting: VPDB was originally used for mineral carbonates, and VSMOW was used as a reference for all other materials (e.g., phosphates, water). VSMOW was used in this study to allow for data comparison with published marine mammal literature.

### 2.6 Data Analysis

All data analyses were conducted using R (v4.4.1) [46]. For each isotope and sample, the paired difference (Δ) between treatments was evaluated by subtracting the isotope value of the treated sub‐sample from the isotope value of the untreated sub‐sample. Δmean or Δmedian, referring to the mean and median, respectively, of these paired differences, were calculated for the following comparisons:

– Removal of structural carbonate (collagen extraction):
  ○ HCl (collagen)/no treatment (bulk dentine) (*n* = 75)
– Removal of collagenous organic matrix (hydroxyapatite extraction):
  ○ H_2_O_2_ (hydroxyapatite)/no treatment (bulk dentine) (*n* = 16)
  ○ Plasma‐ashing (hydroxyapatite)/no treatment (bulk dentine) (*n* = 16)
– Untreated dentine powder drying:
  ○ Dried at 100°C/dried at 80°C (*n* = 10)
  ○ Not dried/dried at 80°C (*n* = 10)

To estimate the potential impact of treatments on isotope values, paired *t*‐tests were performed if the differences between pairs were normally distributed (evaluated using the Shapiro–Wilk test and visual verification of normality histograms and Q‐Q plots, Figures S1–S3). If the distribution of paired differences was not normal, the median of the differences was estimated with the non‐parametric Wilcoxon's signed rank test [47].

The following R packages were used for the creation of visualisation plots: colorspace [48], cowplot [49], ggdist [50], ggplot2 [51], ggpubr [52], patchwork [53], prismatic [54] and raincloudplots [55].

## 3 Results

### 3.1 Removal of Structural Carbonate (Collagen Analysis, δ^13^C and δ^15^N)

**FIGURE 1:**
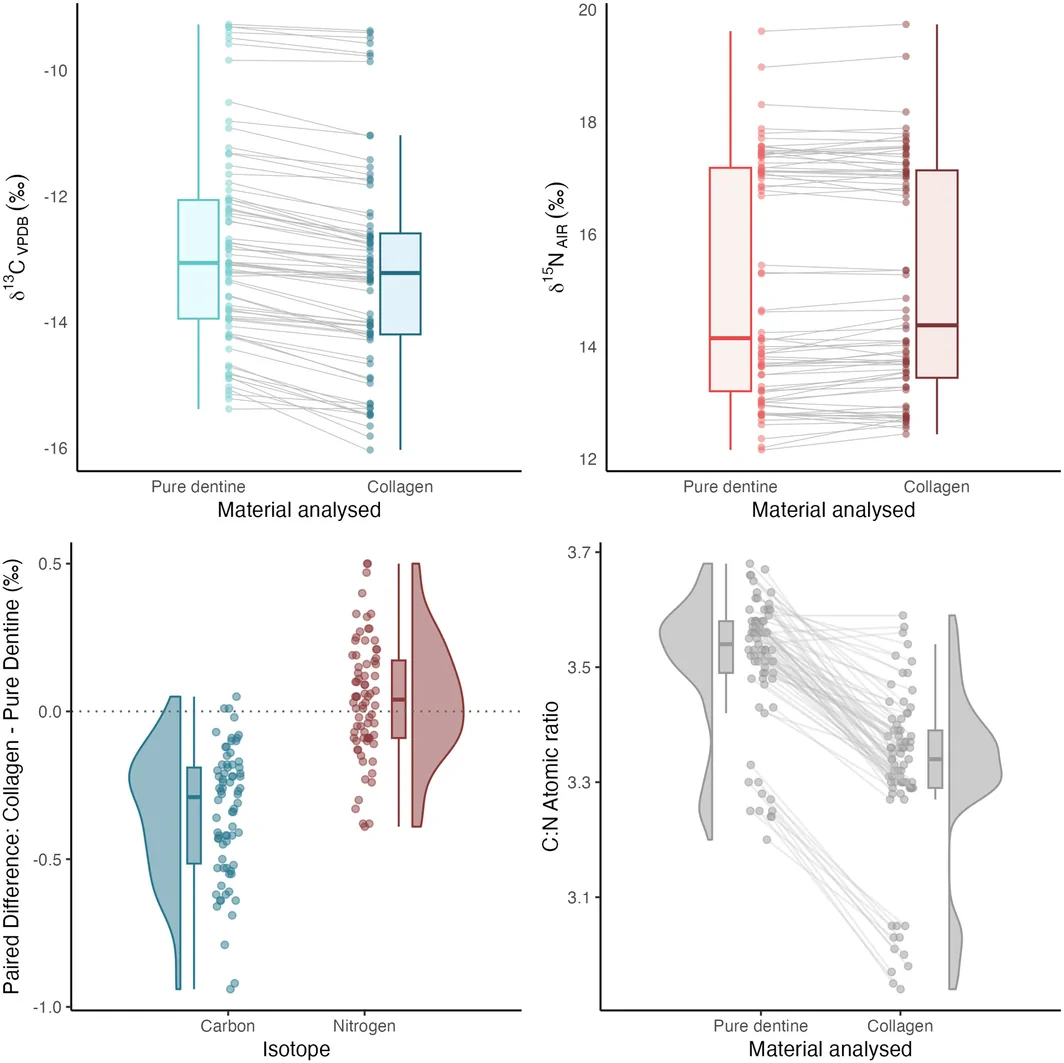
Paired comparison of δ^13^C (top left) and δ^15^N (top right) of pure dentine and collagen. Paired difference for δ^13^C and δ^15^N isotopes (bottom left). Paired comparison of C:N atomic ratio between collagen (HCl‐treated) and untreated dentine samples (bottom right).

Despite careful pipetting of the supernatant during HCl treatments, decalcification of the structural carbonate led to a mean sample loss of 96.6% ± 2.0%. Nonetheless, the collagen obtained was sufficient to obtain isotope values for most analyses (except for collagen samples weighing less than 0.1 mg (*n* = 5)) and the C:N atomic ratio (3.32 ± 0.15) was always within the published expected range of unaltered collagen (2.9–3.6 [56]). The C:N atomic ratio of untreated powder was also within the range of accepted collagen values (3.51 ± 0.12) and was significantly higher than that of the extracted collagen (*p* < 0.05, Δmean = 0.20, 95% CI: 0.18, 0.22) (Figure 1).

The isotope difference between pairs of treated and untreated samples varied by element (Figure 1). δ^13^C was lower in extracted collagen samples (*p* < 0.05, Δmedian = −0.34‰, 95% CI: −0.39, −0.28), whereas δ^15^N was slightly higher (*p* < 0.05, Δmean = 0.05‰, 95% CI: 0, 0.09). HCl treatments led to a δ^13^C decrease in all collagen samples, but the magnitude of the decrease varied at the individual level, from −0.14‰ to −0.54‰, compared with untreated dentine (Figure S4).

### 3.2 Removal of the Collagenous Organic Matrix (Hydroxyapatite Analysis, δ^13^C and δ^18^O)

**FIGURE 2:**
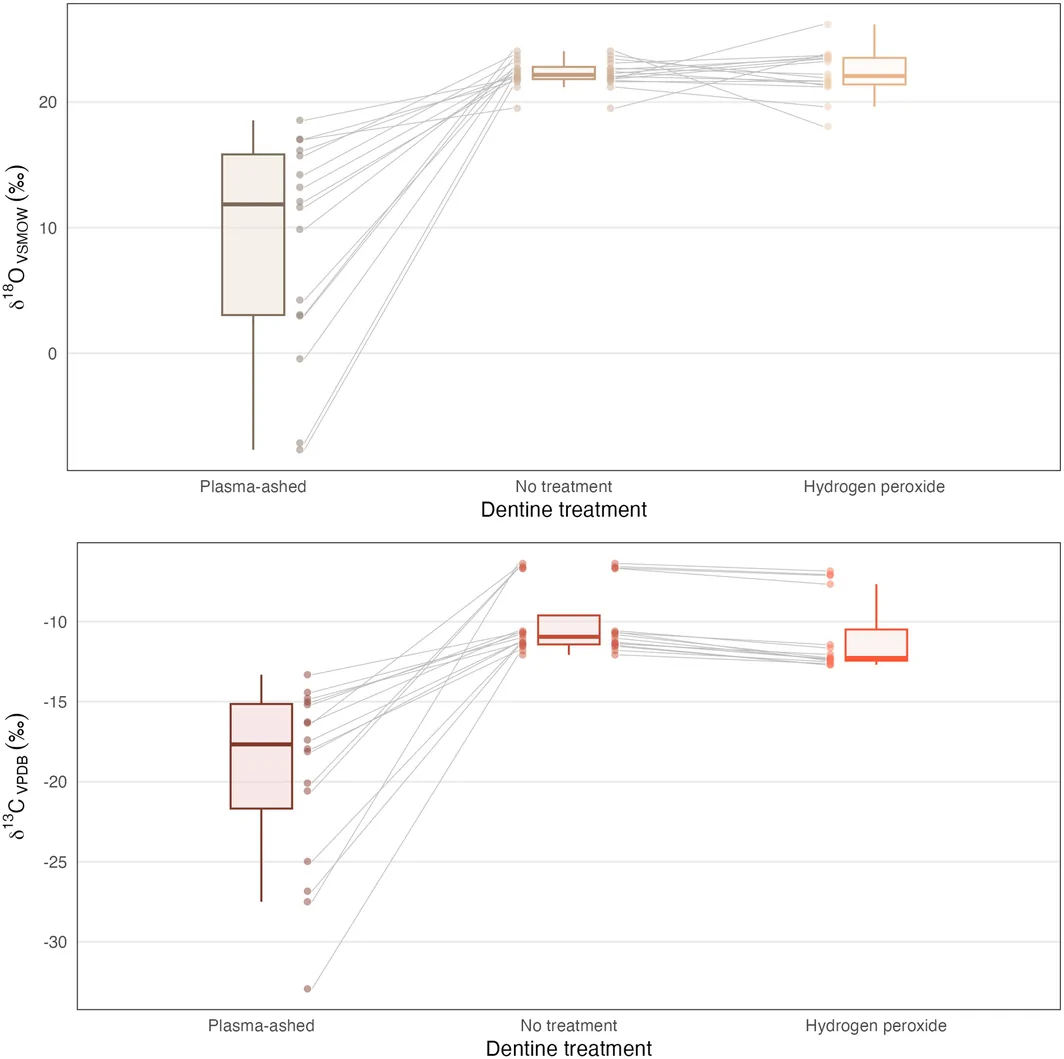
Paired comparison of δ^18^O (top) and δ^13^C (bottom) between untreated dentine powder samples and either plasma‐ashed or H_2_O_2_ treated dentine.

**FIGURE 3:**
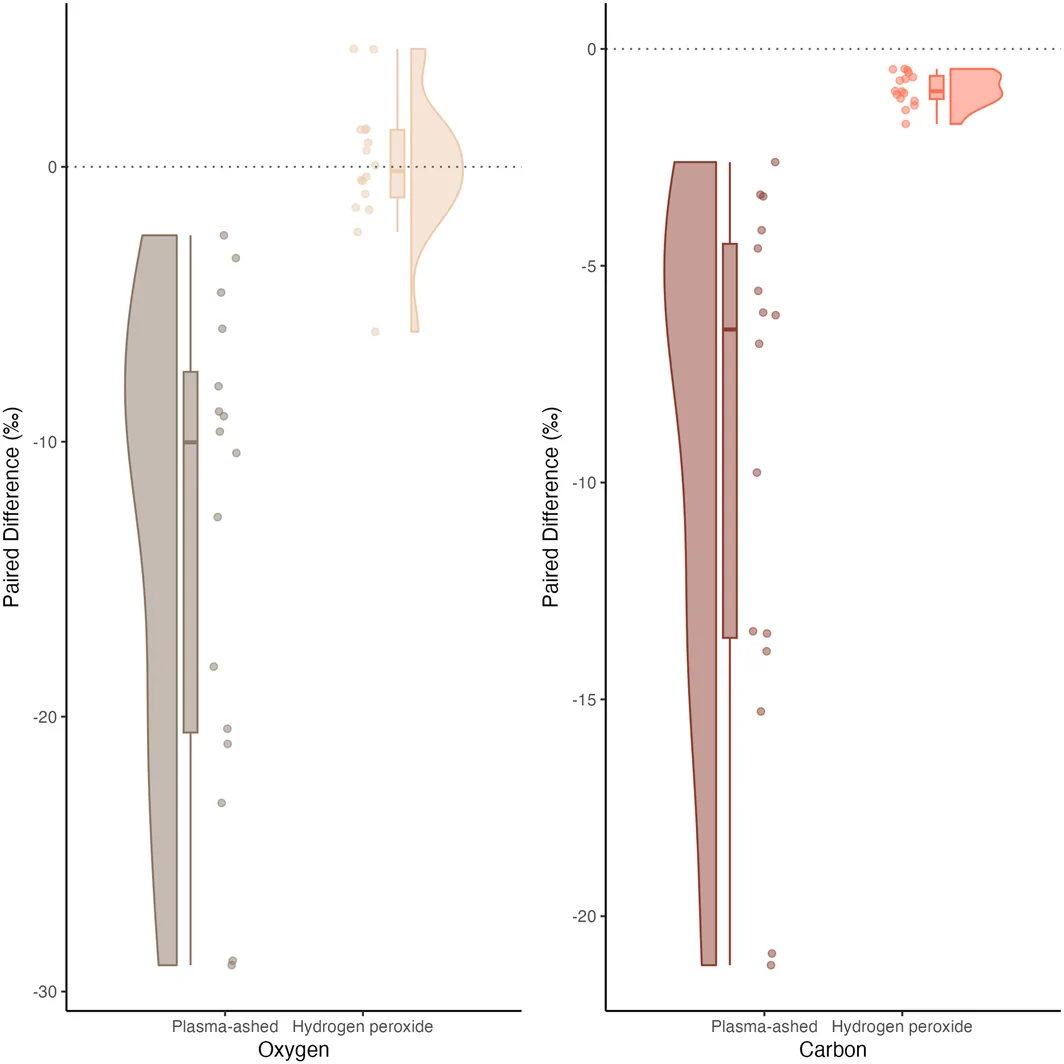
Paired difference for δ^18^O (left) and δ^13^C (right) between treated (plasma‐ashed or H_2_O_2_) and untreated dentine samples.

The impact of organic matrix removal from powdered dentine samples on isotope ratios differed widely across isotopes and treatment methods. The plasma‐ashing process lasted a total of 18 ± 3 h for powdered sub‐samples, after which their weight stopped decreasing, leading to a mean sample loss of 24.4% ± 1.6%. Plasma‐ashing significantly decreased both δ^13^C (*p* < 0.05, Δmedian = −9.04‰, 95% CI: −13.36, −5.13) and δ^18^O (*p* < 0.05, Δmean = −13.48‰, 95% CI: −18.16, −8.80) values relative to untreated dentine (Figures 2 and 3). Although δ^13^C and δ^18^O values always decreased under this treatment, there was substantial variability in the magnitude of difference between each paired sample, as highlighted by the wide confidence intervals for both isotope measures.

H_2_O_2_ treatment resulted in a mean sample loss of 27.8% ± 1.6%. H_2_O_2_ treatment slightly but significantly decreased δ^13^C values (*p* < 0.05, Δmean = −0.93‰, 95% CI: −1.13, −0.73), while the impact on δ^18^O values was variable across paired samples and did not show a clear trend (*p* = 0.97, Δmean = 0.03‰, 95% CI: −1.29, 1.34) (Figures 2 and 3).

Organic matrix removal tests showed nitrogen (wt % and mg) was undetectable in a subset of five samples treated with plasma‐ashing and H_2_O_2_ compared with untreated samples, confirming effective removal of collagen from dentine (Table 3). Unexpectedly, plasma‐ashed samples also lost most of their detectable carbon during treatment.

**TABLE 3:** δ^13^C and δ^15^N results for organic matrix removal in a subset (*n* = 5) of treated and untreated samples. Successful organic matrix removal (undetectable nitrogen) was observed after both treatments but carbon isotope ratios were altered during the plasma‐ashing process. Values are flagged in red if not enough carbon or nitrogen was detectable in the sample by the instrument to obtain a trustworthy δ^13^C or δ^15^N value. One sample (ID01) had enough dentine powder for all three combinations; other samples could only support two combinations.

Sample ID	Treatment	δ^13^C	δ^15^N	mg C	mg N	wt% C	wt% N	C:N ratio
01	No treatment	−9.28	17.18	0.149	0.049	11.13	3.63	3.57
01	Plasma‐ashing	− 9.00	0.41	0.017	0.005	2.06	0.65	3.72
01	H_2_0_2_	−10.61	15.85	0.051	0.012	4.60	1.08	4.98
02	No treatment	−12.73	17.34	0.155	0.051	10.77	3.54	3.55
02	H_2_0_2_	−14.11	16.92	0.064	0.015	4.50	1.07	4.90
03	No treatment	−9.53	17.20	0.138	0.045	10.95	3.57	3.58
03	Plasma‐ashing	− 8.45	− 0.64	0.016	0.005	2.17	0.67	3.80
04	No treatment	−9.47	17.15	0.153	0.049	11.21	3.60	3.63
04	Plasma‐ashing	− 9.17	− 2.35	0.014	0.004	2.83	0.89	3.71
05	Plasma‐ashing	− 9.35	2.61	0.016	0.004	2.45	0.66	4.33
05	H_2_0_2_	−10.93	16.42	0.056	0.013	5.00	1.20	4.85

### 3.3 Dentine Powder Drying (δ^13^C and δ^18^O)

**FIGURE 4:**
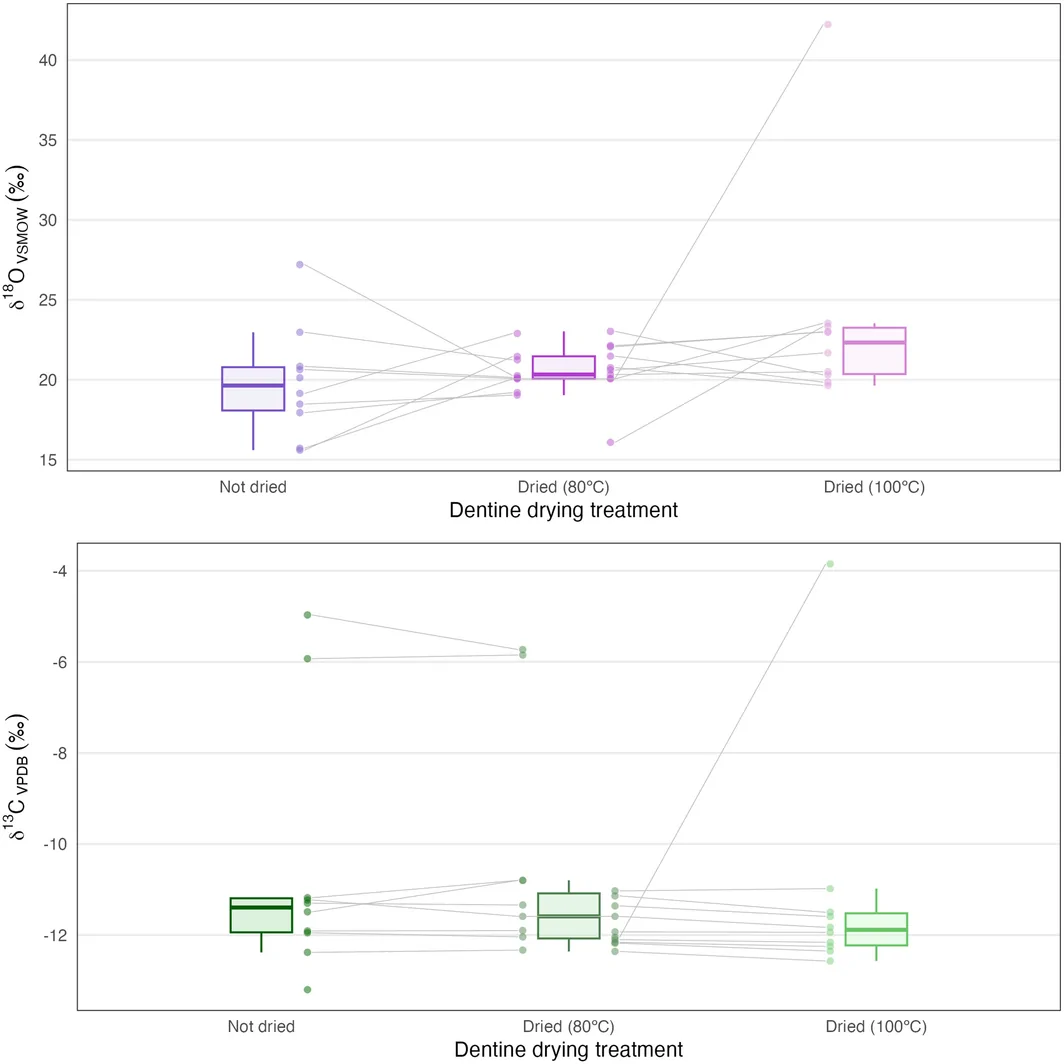
Paired comparison of δ^18^O (top) and δ^13^C (bottom) between dentine powder samples dried at 80°C and dentine either dried at 100°C or not dried.

**FIGURE 5:**
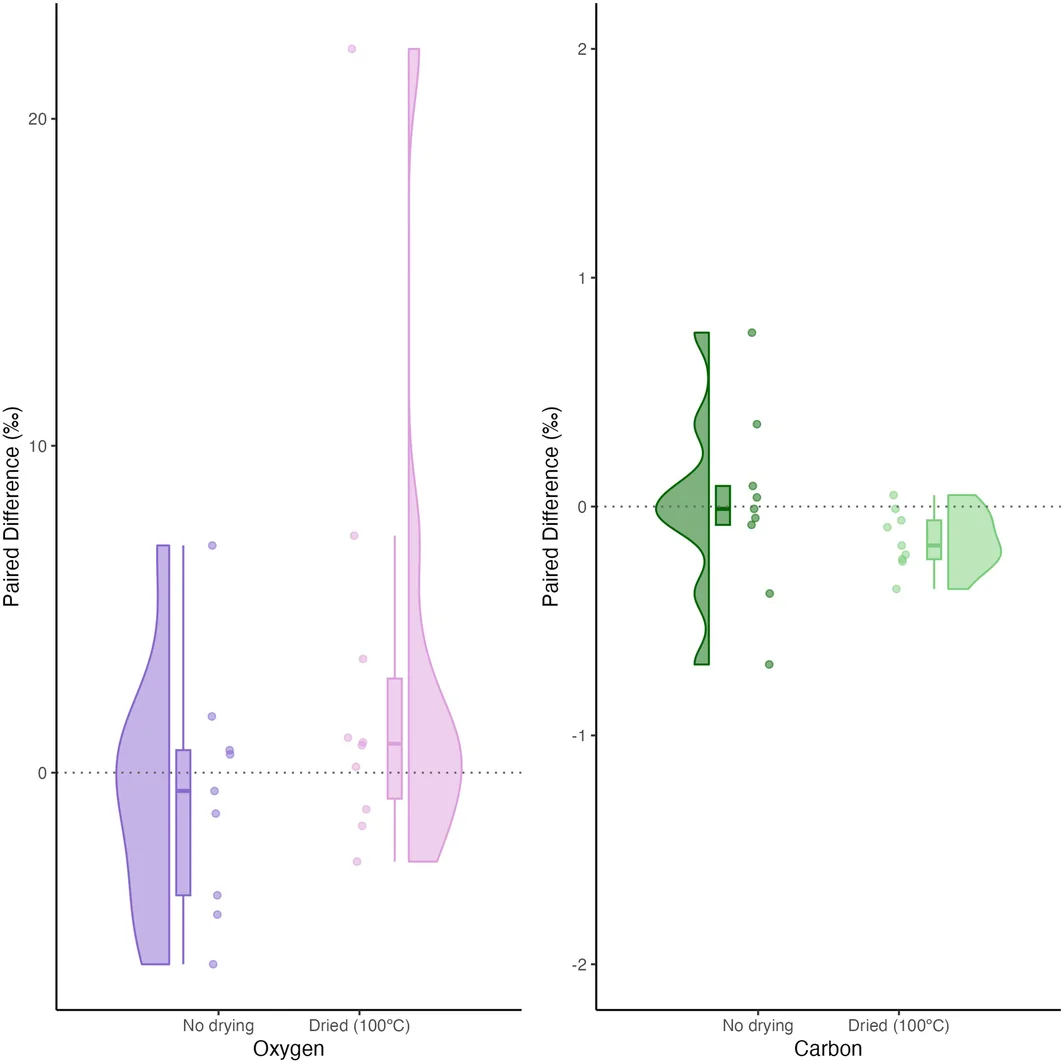
Paired difference for δ^18^O (left) and δ^13^C (right) between dentine samples dried at 100°C or not dried, and the control (dried at 80°C).

Differences between no drying and drying at 80°C were non‐significant (δ^13^C: *p* = 0.98, Δmean = 0‰, 95% CI: −0.31, 0.32; δ^18^O: *p* = 0.63, Δmean = −0.65‰, 95% CI: −3.59, 2.26). Similarly, there was no significant difference between drying at 100°C and at 80°C across δ^13^C and δ^18^O isotopes (δ^13^C: *p* = 0.13, Δmedian = −0.12‰, 95% CI: −0.23, 3.98; δ^18^O: *p* = 0.38, Δmedian = −0.96‰, 95% CI: −0.90, 10.26). No drying and drying at 100°C resulted in a greater variability of isotope ratios for δ^18^O compared with the 80°C baseline (Figures 4 and 5).

## 4 Discussion

### 4.1 Removal of Structural Carbonate (Collagen Analysis, δ^13^C and δ^15^N)

Our results, which demonstrate that HCl treatment has predicted impacts on δ^13^C and C:N atomic ratio and minimal impacts on δ^15^N, support using HCl to remove structural carbonate before δ^13^C and δ^15^N stable isotope analysis of dentine collagen. Although untreated dentine already had a C:N atomic ratio within the collagen range [56], the lowering of that ratio in treated samples was expected given the removal of inorganic carbon (carbonates) by the treatment and indicates successful removal of the target carbonate mineral fraction and retention of the protein [17]. The significant −0.34‰ δ^13^C decrease in collagen samples after HCl treatment was also anticipated because δ^13^C in collagen is isotopically lighter (more ^12^C than ^13^C) than δ^13^C in structural carbonate [57].

Unintended effects on δ^15^N have often been cited as a reason to avoid acidification, particularly when both δ^13^C and δ^15^N are measured consecutively on a single sample. Although we measured a consistent δ^15^N difference between treated and untreated samples, it was minimal (0.05‰), within measurement error (SD = 0.14‰), and of small magnitude with respect to ecologically relevant differences (e.g., trophic‐level differences are between 1‰ and 3‰ [58]). Other studies similarly found no difference in δ^15^N between paired samples when conducting collagen extraction treatments on marine mammal dentine [6, 17, 18]. Given that most of the nitrogen in dentine is found in collagen, removing structural carbonate should not have a significant effect on δ^15^N, consistent with our findings.

Based on these results, HCl treatment would be recommended prior to analysis when only one aliquot is available (requiring the simultaneous analysis of δ^13^C and δ^15^N) to remove the ^13^C‐enriched carbonates. This result matches that from a recent study on treatments of killer whale bone prior to stable isotope analysis [59], but disagrees with several other studies on marine mammals [6, 17, 18, 60], which did not find a difference in δ^13^C after treatment. Individual variability as well as differences across species could explain these opposing results. Indeed, the isotopic composition of collagen and carbonates is partly controlled by the quantity of proteins, lipids and carbohydrates present in the diet [11]. For example, the collagen δ^13^C of a species with a proteinaceous diet would be more similar to its carbonate δ^13^C compared with that of an animal with a diet with a large lipid content [11], which would modify the detectable difference between paired samples in treatment tests. Killer whales are known to predate on a wide range of prey taxa across their global range [61]. The variability in the magnitude of the δ^13^C decrease seen in this study could originate from population or individual‐specific differences, which could alter isotopic results, but this cannot be confirmed until the diet of these populations is better assessed. Variable results among studies comparing stable isotopes of bulk and acid‐treated dentine could similarly be due, at least in part, to the dietary differences among the different study species.

Therefore, we suggest that testing of different treatments should always be conducted prior to analysis and recommend that the testing dataset should be representative of the entire dataset. If treatment is found to be necessary and some samples are too small to be treated, treatment tests should be conducted, and a correction factor (here, −0.34‰ for δ^13^C) should be estimated and applied to the isotope ratios of the untreated samples. Studies analysing other taxa and samples with lower carbonate content (e.g., whole marine microorganism, arthropod muscles) found that acidification treatments led to significant, undesirable changes in δ^15^N [15, 16]. This was not observed with dentine samples in this study, but if the changes in δ^15^N were significant, untreated aliquots should be used for nitrogen analysis.

### 4.2 Removal of the Collagenous Organic Matrix (Hydroxyapatite Analysis, δ^13^C and δ^18^O)

Treatments to remove the collagenous organic matrix from killer whale dentine samples prior to δ^13^C and δ^18^O stable isotope analysis successfully removed organic material but had large unintended consequences. Mass loss during organic matrix removal with plasma‐ashing and H_2_O_2_ was within the expected collagen proportion in dentine [57]. However, plasma‐ashing had the undesirable effect of removing or altering the carbonate carbon as well, leading to large changes in the δ^13^C value of the analysed sub‐samples. Organic matrix removal by plasma‐ashing takes place when a plasma composed of excited oxygen molecules comes in contact with the dentine powder, thus oxidising the organic matrix into CO_2_ and H_2_O [62]. While this low‐temperature, contamination‐free process should preserve the bioapatite structure [36], these results suggest that this method did not maintain the original characteristics of hydroxyapatite. We hypothesise that unknown and unpredictable chemical reactions occurred during the plasma‐ashing process, modifying the chemistry of the carbonates prior to analysis. Such undesirable reactions have previously been reported in other studies, and include breakage of light oxygen isotopes from the carbonate (CO_3_^−^) molecule [62] or isotopic exchange with atmospheric CO_2_ [63]. Carbonisation of samples during the ashing process is another possible source of material alteration, but this can be easily noticed by a powder colour change [34], which was not observed in this study. The large negative shift (> 8‰) in isotope values for both δ^13^C and δ^18^O, resulting in isotope values falling outside of the documented cetacean dentine range [5, 8], as well as the reduction in carbon wt% and mg measured by the elemental analyser, indicate that carbonate structure was affected and plasma‐ashing is therefore not a recommended treatment for dentine.

Hydrogen peroxide treatments removed most of the organic matter, and a positive δ^13^C isotopic shift due to the removal of ^13^C‐depleted collagen [14] was therefore expected. However, we observed the opposite effect (a small decrease in δ^13^C in treated samples). Negative δ^13^C isotopic shifts following H_2_O_2_ treatment have also been reported in other studies [28, 64]. A possible explanation for this result is that during phosphoric acid exposure, new carbonates are produced from the CO_2_ present in solution and have lower δ^13^C values (originating from other particles than the dentine structural carbonate, e.g., organic matrix, impurities) [31, 33]. Indeed, treatments such as H_2_O_2_ could have the opposite effect on isotopic values by increasing the reactivity of residual organic material while failing to remove it [33]. A negative shift in δ^13^C could also be explained by removal of inorganic carbon (^13^C‐enriched carbonates) during H_2_O_2_ treatment caused by organic acid production [31, 63]. Finally, the presence of secondary carbonates due to diagenetic alteration of fossil specimens has also been found to result in an isotopic shift [27], but this should not be the case here because samples were modern. The H_2_O_2_ treatment effect on δ^18^O was inconclusive but increased the isotopic range of the treated samples compared with untreated dentine. While some studies have shown that H_2_O_2_ treatment can increase δ^18^O [27, 31, 64], others did not find a difference [28, 33] or found a decrease following treatment [34]. These results suggest that H_2_O_2_ is not recommended for removal of organic material in dentine.

If treatments for organic matrix removal before carbonate isotope analysis are inconclusive, analyses can be undertaken with untreated samples instead [5, 31]. Indeed, phosphoric acid normally does not interact with collagen in solution, so the analysis of structural carbonates should not be impacted by the presence of organic matrix [65].

### 4.3 Dentine Powder Drying (δ^13^C and δ^18^O)

Drying untreated powdered samples overnight at 80°C was the most reliable preparation method for δ^18^O measurements of dentine carbonate. High δ^18^O variability in non‐dried samples compared with 80°C‐dried samples could be explained by a variable amount of residual water present in dentine samples [5] or atmospheric conditions at the time of drilling. Indeed, the microdrilling process takes a couple of hours and powder is exposed to the surrounding air; humidity in a room could therefore affect the amount of water adsorbed by the powder prior to being transferred into the vial for analysis. The hygroscopic nature of dentine [66] might prevent complete removal of all residual or adsorbed water when drying at 80°C and might lead to lower δ^18^O values, but drying at 100°C also increased the variability of the data and led to some extreme values, suggesting this procedure might be best avoided because there is a risk of overheating which could modify the resulting isotopic value of a sample [67]. Therefore, we suggest oven‐drying samples at 80°C when analysing structural carbonates from killer whale dentine and recommend testing with a subset of the samples prior to analysis.

## 5 Conclusion

Treatments, sample preparation [47], instrument and solvent variability [30], and potential population variability could all influence the overall individual isotopic value, so care is needed when interpreting and particularly when comparing with previously published data. We recommend that (i) HCl treatment should be undertaken prior to δ^13^C and δ^15^N analysis of killer whale dentine collagen, (ii) if removal of organic matter is necessary, plasma‐ashing should be avoided, (iii) no treatment should be conducted on killer whale dentine powder other than overnight drying at 80°C for δ^13^C and δ^18^O analysis of the hydroxyapatite and (iv) where material is insufficient for carbonate removal by HCl decalcification, we suggest testing and treating a subset of the samples to apply a correction factor. We emphasise that reporting test outcomes is essential to save time and resources, and to successfully compare stable isotope studies within and across species.

## Author Contributions

**Maeva Terrapon:** conceptualization, investigation, funding acquisition, writing – original draft, methodology, visualization, writing – review and editing, data curation, formal analysis. **Philip S. Hammond:** conceptualization, supervision, funding acquisition, writing – review and editing, validation. **Rona A. R. McGill:** supervision, data curation, writing – review and editing, methodology, validation. **Jason Newton:** supervision, methodology, validation, writing – review and editing. **Julie Dougans:** methodology, writing – review and editing, data curation. **Cory J. D. Matthews:** conceptualization, validation, writing – review and editing, methodology, supervision. **Sascha K. Hooker:** conceptualization, funding acquisition, writing – review and editing, supervision, validation.

## Funding

This work was supported by National Environment Isotope Facility, 2429.1021; School of Biology Postgraduate Scholarship; AD Links Scholarship.

## Supporting information


**Figure S1:** Tests for normality. Histogram and normal Q‐Q plot of (i) top, the paired differences in carbon isotope between HCl‐treated and untreated dentine powder and (ii) bottom, the paired differences in nitrogen isotope between HCl‐treated and untreated dentine powder.
**Figure S2:** Histogram and normal Q‐Q plot of (i) first line, the paired differences in carbon isotope between H_2_O_2_‐treated and untreated dentine powder, (ii) second line, the paired differences in oxygen isotope between H_2_O_2_‐treated and untreated dentine powder, (iii) third line, the paired differences in carbon isotope between plasma‐ashed and untreated dentine powder and (iv) second line, the paired differences in oxygen isotope between plasma‐ashed and untreated dentine powder.
**Figure S3:** Histogram and normal Q‐Q plot of (i) first line, the paired differences in carbon isotope between not‐dried and 80°C‐dried dentine powder, (ii) second line, the paired differences in oxygen isotope between not‐dried and 80°C‐dried dentine powder, (iii) third line, the paired differences in carbon isotope between 100°C‐dried and 80°C‐dried dentine powder and (iv) second line, the paired differences in oxygen isotope between 100°C‐dried and 80°C‐dried dentine powder.
**Figure S4:** rcm70176‐sup‐0001‐Figures_S1‐S4.docx.*.* Paired comparison of δ^13^C (left) and δ^15^N (right) of untreated dentine and collagen, coloured by individuals.

## Data Availability

The data that support the findings of this study are available from the corresponding author upon reasonable request.
