# Supplementary material for: Stable Isotope Analysis (δ13C, δ15N and δ18O) of Killer Whale Dentine: Evaluating Treatment Methods and Establishing Best Practices
Source: Rapid Commun Mass Spectrom. 2026 Sep 15;40(23):e70176. doi: 10.1002/rcm.70176 (PMC13578864; doi:10.1002/rcm.70176)
Supplement: Supplementary file 1 [file RCM-40-e70176-s001.docx]

**Supporting information**

*Figure 1.* Tests for normality. Histogram and normal Q-Q plot of i) top, the paired differences in carbon isotope between HCl-treated and untreated dentine powder and ii) bottom, the paired differences in nitrogen isotope between HCl-treated and untreated dentine powder.

*Figure 2.* Histogram and normal Q-Q plot of i) 1^st^ line, the paired differences in carbon isotope between H_2_O_2_-treated and untreated dentine powder, ii) 2^nd^ line, the paired differences in oxygen isotope between H_2_O_2_-treated and untreated dentine powder, iii) 3^rd^ line, the paired differences in carbon isotope between plasma-ashed and untreated dentine powder, and iv) 2^nd^ line, the paired differences in oxygen isotope between plasma-ashed and untreated dentine powder.

*Figure 3.* Histogram and normal Q-Q plot of i) 1^st^ line, the paired differences in carbon isotope between not-dried and 80 ºC-dried dentine powder, ii) 2^nd^ line, the paired differences in oxygen isotope between not-dried and 80 ºC-dried dentine powder, iii) 3^rd^ line, the paired differences in carbon isotope between 100 ºC-dried and 80 ºC-dried dentine powder, and iv) 2^nd^ line, the paired differences in oxygen isotope between 100 ºC-dried and 80 ºC-dried dentine powder.

*Figure 4.* Paired comparison of δ^13^C (left) and δ^15^N (right) of untreated dentine and collagen, coloured by individuals.
